# *Helicobacter pylori* Infection in Children: A Possible Reason for Headache?

**DOI:** 10.3390/diagnostics13071293

**Published:** 2023-03-29

**Authors:** Ancuta Lupu, Cristina Gavrilovici, Vasile Valeriu Lupu, Anca Lavinia Cianga, Andrei Tudor Cernomaz, Iuliana Magdalena Starcea, Cristina Maria Mihai, Elena Tarca, Adriana Mocanu, Silvia Fotea

**Affiliations:** 1 Pediatrics, “Grigore T. Popa” University of Medicine and Pharmacy, 700115 Iasi, Romania; 2 III-rd Medical Department, “Grigore T. Popa” University of Medicine and Pharmacy, 700115 Iasi, Romania; 3 Pediatrics, Faculty of General Medicine, Ovidius University, 900470 Constanta, Romania; 4 Department of Surgery II—Pediatric Surgery, “Grigore T. Popa” University of Medicine and Pharmacy, 700115 Iasi, Romania; 5 Medical Department, Faculty of Medicine and Pharmacy, “Dunarea de Jos” University of Galati, 800008 Galati, Romania

**Keywords:** headache, *Helicobacter pylori*, children, migraine

## Abstract

(1) Background: The correlation between infection with *Helicobacter pylori* (*H. pylori*) and headache has been argued and explored for a long time, but a clear association between the simultaneous presence of the two in children has not been established yet. In this study, we aimed to explore this relationship in children from the Northeast region of Romania. (2) Methods: A retrospective study exploring the correlation between children having *H. pylori* infection and headache or migraine was conducted on a batch of 1757 children, hospitalized over 3 years in a pediatric gastroenterology department in Northeast Romania. (3) Results: A total of 130 children of both sexes had headache. From 130 children, 54 children (41.5%) also presented *H. pylori* infection. A significant association between headache and *H. pylori* infection (χ2; *p* < 0.01) was noticed. (4) Conclusions: More studies are needed on this relationship, and we emphasize the importance of further analyses, as they present great clinical importance for both prompt diagnosis and treatment.

## 1. Introduction

Headache represents a common complaint among the pediatric population, which, as well as in adults, is frequently underdiagnosed, although it affects the quality of life. The prevalence of migraine, one of the most common types of primary headache, is estimated at approximately 9% among the pediatric population, and a perpetual increase in its incidence has been registered over the last three decades. Among the indicative factors of an increased incidence of headache, Anttila et al. mention sleep deprivation, an increase in the use of information technology, and soft drink consumption [1]. In children, migraine and tension-type headache usually occur simultaneously with a mixed symptomatology [2]. They are usually considered to be self-limited conditions, but they can persist from childhood to adulthood, affecting the quality of life [3]. Being a burdensome condition, headache plays an important role in mental and physical health, and in children, it can impair school performance or lead to social isolation [4,5]. Thus, headache can interfere negatively with the entire education path in childhood and adolescence [6]. In children, headache can present through neurobehavioral symptoms such as agitation, sleep disturbances, irritability, and trouble concentrating [7]. Various factors such as sleep disorders, genetics, environmental factors such as humidity, light, or noise, severe trauma, and menstruation have been indicated as risk factors and possible triggers of migraine headaches [8]. The literature suggests that there may be a significant connection between *Helicobacter pylori* (*H. pylori*) infection and headaches. However, though this association has been explored in adults, there is still a significant gap in data regarding this issue in the pediatric population [9].

*H. pylori* is a gram-negative, microaerophilic, spiral bacterium with increased motility as a result of the presence of multiple unipolar flagella [10]. It generates urease and colonizes the mucus layer adjacent to the gastric mucosa, usually being responsible for gastrointestinal impairments such as chronic active gastroenteritis, infection, gastric and duodenal ulcer, and, more rarely, stomach cancer [11]. The bacterium possesses adaptive characteristics that allows the body’s survival in an acidic environment. It produces urease that consequently converts urea into bicarbonate and ammonium and leads to the neutralization of the gastric acid [12]. However, numerous studies claim that infection with *H. pylori* may be the result of various extra-digestive conditions such as neurological, cardiovascular, metabolic, hematologic, ocular, or dermatological ones.

Along with various extra-digestive impairments, the relationship between the infection with *H. pylori* and neurological manifestations such as mild cognitive impairment, migraine, or Alzheimer’s disease have been extensively studied, but there are no clear results concerning the pathophysiology of the process [13]. However, it was shown that the systemic effects of the infection with *H. pylori* are the result of the modulation of the gut–brain axis (GBA), which consists of a two-way signaling pathway between the gastro-intestinal tract (GIT) and the brain, and which plays a pivotal role in infections and additional clinical outcomes [14,15,16].

Among the stated hypotheses, *H. pylori* infection may trigger a host immune response to the presence of bacteria and a consequent release of vasoactive substances [17]. The pathological process encompasses immunological events such as migration of lymphocytic, monocytic, and neutrophilic invasion into the gastric mucosa and submucosa, along with the release of chemokines or pro-inflammatory cytokines such as IL-6, IL-1β, TNF-α, or IL-8 at the site of infection [18,19]. Moreover, *H. pylori* infection disturbs the balance in the microbiota, influences the host–pathogen interaction, and plays an important role in the modulation of the gastric microenvironment, thus causing changes in homeostasis [20,21].

*H. pylori* type I cagA-positive strains are also thought to have the ability to induce a significant release of proinflammatory substances by the gastric mucosa, leading consequently to systemic vasospasms [22]. As described in the literature, for patients with *H. pylori* infection who complain of headache, bacteria eradication might improve the symptoms or reduce the migraine-related disability level [19].

There exist multiple pharmaceutical treatment plans for managing both digestive and extra-digestive infections and diseases caused by *H. pylori*. Timely and precise identification of *H. pylori* plays a critical role in the effective treatment and eradication of the bacterium. Due to *H. pylori*’s specific localization in the gastric mucus layer, pharmacotherapy must be effective in penetrating this layer to prevent *H. pylori* colonization. As such, the medication used must be able to penetrate the gastric mucosal layer [23].

The data on children in Romania regarding *H. pylori* infection reveals a declining trend that might be the result of improving socio-economic conditions [24]. However, Yuan et al. highlighted in their review that despite advances in medical science, *H. pylori* infection continues to exhibit a high incidence rate among children worldwide, emphasizing the significance of this infection [25].

Although the data regarding the link between *H. pylori* infection and headache have been explored for many years, and the results of this association are controversial, we present the results we obtained on a pediatric population in Northeast Romania.

## 2. Materials and Methods

We performed a retrospective study on 1757 children, hospitalized over 3 years in a pediatric gastroenterology department in “St. Maria” Emergency Hospital for Children in Iasi, Romania, complaining of symptoms suggestive of gastric or duodenal ulcer. Thus, according to Jones et al.’s recommendations from 2017 [26], all the 1757 patients underwent superior digestive endoscopy. With a treatment that was likely to be offered, biopsies and cultures were taken for the examined patients. The diagnostic of infection with *H. pylori* was established by having *H. pylori*-positive gastritis on histopathology examination, along with positive cultures. For these patients, we focused on the association between the *Helicobacter pylori* infection and the presence of headache/migraine. Out of the 1757 patients of both sexes, we selected based on the anamnestic findings those who complained of migraine or headache at admission. To assess the importance of headache, the Migraine Disability Assessment Test (MIDAS) along with the Visual Analogue Scale (VAS) were utilized.

We excluded children who previously received eradication treatment of *H. pylori*, those who previously had treatment with acetaminophen or antibiotics, children with evidence of bleeding of the gastro-intestinal tract at endoscopy, those who complained of headache or migraine during previous hospitalizations or had a medical past history of headache/migraine, patients with gastrointestinal disorders known to be associated with headache such as inflammatory bowel syndrome, celiac disease, or functional abdominal pain, and patients with a history of drug use, including H2 blockers, antibiotics, or proton pump inhibitors, within 4 weeks [27,28,29].

Based on the available information in the literature, migraine was defined as the presence of severe and recurrent headache attacks, along with neurological and autonomic symptoms. The diagnostic criteria for pediatric migraine were realized according to the second edition of the International Classification of Headache Disorders (ICHD-2) [30].

All patients enrolled underwent upper gastrointestinal endoscopic examination with intravenous sedation, and video pediatric gastroduodenoscopes from Pentax and Olympus were used. For children under 10 years old, the procedure was performed under general anesthesia. Biopsies were collected from the antrum and gastric corpus during endoscopy for rapid urease testing and histological and bacteriological examination [27,28,29].

Informed consent was taken from all caregivers, and the study was approved by the “St. Mary” Emergency Hospital for Children Ethics Committee’s (no.31490/29.10.2021).

Data were extracted from the hospital database, patient observation charts, endoscopy results, and discharge papers. IBM SPSS 17.0 platform, GraphPad Prism, and Microsoft Excel were used to analyze the data.

## 3. Results

From the 1757 patients, 542 had infection with *H. pylori*. We reported the structure of the study group in Table 1.

The main symptoms that led to admission to the pediatric gastroenterology clinic and that later resulted in the diagnosis of gastritis were represented in order of frequency by: abdominal pain in 1664 cases (94.7%), nausea in 668 cases (38.0%), vomiting in 468 cases (27.2%), inappetence in 243 cases (13.8%), heartburn in 144 cases (8.2%), headache in 130 cases (7.4%), vertigo in 87 cases (5.0%), constipation in 57 cases (3.2%), abdominal flatulence in 56 cases (6.2%), asthenia in 26 cases (1.5%), and early satiety in 17 cases (1.0%).

Among the non-specific symptoms, we found a strongly significant association between headache and infection with *H. pylori* (χ2; *p* < 0.01) (Table 2). Of the 130 children who had headaches, 54 children (41.5%) were also diagnosed with infection with *H. pylori*.

Important differences between sex-stratified subgroups regarding *H. pylori* infection and headache were also noticed. Our evaluation showed that the prevalence of headache was almost five times higher in girls, out of the total number of 130 cases (83.1% females vs. 16.9% males). (Table 3).

Moreover, regarding the area of living, we noticed a higher number of children from rural areas—104 children (80%), compared to 26 children (20%) from urban areas—among all cases with headache.

**Figure 1:**
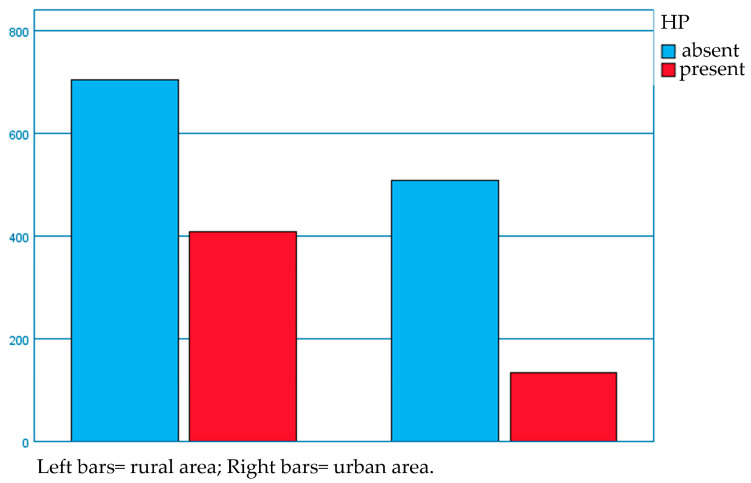
Environmental distribution of patients with or without infection with *H. pylori*.

Subsequently, we evaluated the environmental distribution of *H. pylori* (+) and (−) patients. The distribution of children with *H. pylori* (+) according to the area of living revealed a frequency of 75.3% in rural environments compared to 24.7% in urban areas (Figure 1).

**Figure 2:**
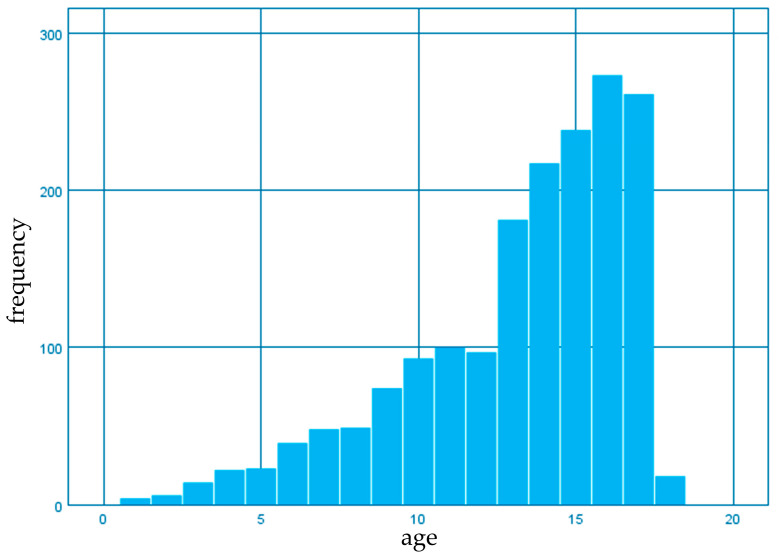
Age distribution of patients with or without infection with *H. pylori*.

In addition, the exploration of the age distribution of all the patients from the initial batch showed a mean value of 13.19 years (SD = +3.501) (Figure 2).

Considering the population analyzed, which is represented by hospital-referred pediatric patients with clinical pictures suggestive of gastritis, there seems to be a significant difference in the odds of having chronic headache complaints between the *H. pylori* positive and negative subgroups: odds ratio = 1.658 (95% confidence interval [CI]: 1.15–2.38)—as indicated by a post-hoc binary logistic regression model including the presence of headache as the dependent variable and *H. pylori* infection status, gender, and living conditions as covariates. The computed power was 77% (given the sample size of 1757 and an effect size of 0.065), and the goodness of fit of the model was deemed low. More data would be useful, but given the retrospective nature of our analysis, no viable solution to enlarge the data pool was identified.

## 4. Discussion

Migraine is a common condition consisting of primary headaches with a prevalence of 15% in Western societies [31]. Secondary headache is frequently reported by patients with various gastrointestinal disorders such as gastroesophageal reflux disease, inflammatory bowel syndrome, constipation, functional abdominal pain, or *H. pylori* infection, but the potential causal link remains unclear [32,33]. However, in recent years, research has focused on the implication of *H. pylori* activity in the pathogenesis of migraine, as this microorganism was identified as a cause for multiple extra digestive manifestations [22]. It has been hypothesized that the recurrence of headaches following *H. pylori* infection may be due to the systemic vasospastic effects of proinflammatory substances that are released by the infected gastric mucosa [34]. Other authors indicate the production of platelet-activating factor in *H. pylori* infections and claim that the migraine attacks may be the result of the high level of serotonin released from platelets [35]. Eradication of *H. pylori* infection resulted in a significant reduction in the intensity, frequency, and duration of migraine attacks [36,37]. However, the studies conducted on the correlation between *H. pylori* infection and headache have provided mixed and controversial results.

Kikui et al. showed in their study that in comparison to non-migraine individuals of similar characteristics, migraine sufferers exhibit a higher prevalence of gastrointestinal comorbidities—specifically, increased odds of irritable bowel syndrome (adjusted odds ratio (95% CI: 3.8 (2.7 to 5.4)), heartburn (3.6 (95% CI 2.8 to 4.7)), gastroesophageal reflux disease (3.5 (2.5, 4.8)), ulcers (3.1 (95% CI 2.0 to 4.8)), frequent diarrhea (3.1 (95% CI 2.3 to 4.1)), and chronic constipation (2.5 (95% CI 1.9 to 3.3)) [38].

Our study found a strongly significant association between headache and infection with *H. pylori* (χ2; *p* < 0.01). Out of the 130 children who complained of headaches, 54 of them (41.5%) also had concomitant *H. pylori* infection. Thereby, a high prevalence of infection with *H. pylori* in patients with headache/migraine is indicated. These results are in agreement with the findings of Cavestro et al., who conducted an impressive cross-sectional study on the relationship between *H. pylori* infection and headache and found a significant association between these two entities (*p* = 0.009) [39]. Moreover, in their case-control study, Yiannopoulou et al. presumed the same association and found that *H. pylori* infection prevalence was significantly higher in 49 patients with headache than in 51 control subjects (*p* = 0.016) [40]. In their study on 70 patients, Hosseinzadeh et al. also showed that the prevalence of migraine was significantly correlated with the IgG and IgM titer against *H. pylori* (*p* ≤ 0.048 and *p* ≤ 0.03, respectively) [41].

In addition, the eradication treatment for *H. pylori* infection proved a beneficial effect on the patients suffering from migraine compared to controls in a study conducted by Tunca et al. [36]. Comparable results were obtained in 2012 by Faraji et al., who showed that their patients with migraine who received *H. pylori* eradication treatment presented a lower headache-related disability level than those in the placebo batch. The mechanisms involved could be linked to oxidative stress and nitric oxide imbalance secondary to acute inflammation caused by *H. pylori* infection [42]. The same improvement was obtained by Karkelis et al., who evaluated a number of 65 children suffering from headache and migraine and discovered that 17 of them had concomitant *H. pylori* infection. After completing the anti-Helicobacter infection therapy, a complete resolution of migraine symptoms was noticed for all the patients in the study [43].

All these studies agree with the results reported by Gasbarrini et al. in 1997 and 1998, who described both an association between *H. pylori* infection and headache, as well as a significant alleviation of the intensity of headache along with the eradication of *H. pylori* infection [44,45]. In their case-control study, Hassan et al. obtained a similar result with a significant prevalence of infection with *H. pylori* in 77 migraine patients (*p* < 0.001, OR = 3.439), but at the same time, they obtained no correlation between *H. pylori* infection and migraine attacks, migraine disability assessment test, or the visual analogue scale. In addition, *H. pylori* infection did not represent a trigger for the migraine attacks or a risk factor for an increased frequency of headache episodes [46].

Su et al. also described in their meta-analysis of five case-control studies that *H. pylori* infection was positive in approximately 45% of patients with migraine compared to a prevalence rate of 33% among healthy controls (OR = 1.92, 95%CI: 1.05–3.51, *p* = 0.001). Moreover, the infection rate of *H. pylori* was higher in Asian patients with migraine, but the same could not be established for European ones (OR = 3.48 and 1.19, respectively) [47].

In contradiction with the results above, Lee et al. found a higher frequency of *H. pylori* infection in patients with migraines or headaches than in the control groups, but no statistical significance was obtained (*p* = 0.51) [48]. In Iran, a study was conducted on 84 patients that revealed a significant correlation between the severity of headache and the IgG antibody. However, there was no statistically significant difference observed in levels of IgG in migraine versus control subjects. On the other hand, they observed a statistical significance in the IgM antibody titer against *H. pylori* among the patients with migraine compared to those in the control batches (*p* = 0.004) [49]. The role of interleukin-10 was also speculated, on one hand because its elevation was associated with both migraine and *H. pylori* infection (cagA-positive strains, in particular), and on the other hand, due to the fact that sumatriptan (5-HT1D receptor agonist) decreases the levels of this cytokine during a migraine attack [50,51].

In another study conducted on 31 children complaining of migraine and abdominal pain, an impressive high prevalence of esophagitis (41.9%), antral gastritis (38.7%), duodenitis (87.1%), and corpus gastritis (51.6%) was found. However, only seven patients out of the total had simultaneous *H. pylori* infection, and no association between migraine and *H. pylori* infection could be made [52]. In the same manner, Ciancarelli et al. evaluated 30 subjects suffering from migraine and found that for only 16.7% of them, the infection with *H. pylori* was confirmed, leading to the absence of a certain association between infection with *H. pylori* and migraine [53].

Interestingly, a retrospective study from Turkey performed on 526 subjects with migraine described that the infection with *H. pylori*, as a chronic infection, can be more aggressive and may represent one of the risk factors of the apparition and development of matter lesions in these patients. Here, Ocal et al. found that white matter lesions (WMLs) were present in 178 (33.8%) *H. pylori*-positive subjects (*p* < 0.05), and more than that, there was a 2.5-fold higher incidence of WMLs on the brain MRIs of migraine patients with *H. pylori* infection [54]. In an impressive cross-sectional study from 2021 that covered 489,753 participants, Welander et al. found a significant association between migraine and *H. pylori* infection when entered separately, but with other gastro-intestinal conditions added to the same adjusted model, the statistical significance could not be validated (OR = 1.34, *p* = 0.024) [55].

Having a multifactorial susceptibility with hormonal, genetic, and environmental factors each playing different, but important roles, headache affects over 17% of females and only 5–8% of males [56]. In our study, we also found that out of the 130 patients who complained of headache, 108 were females (83.1%), and only 22 were males (16.9%). Hormati et al. also described in their research on 341 patients the presence of a higher prevalence of migraine among females (*p* = 0.003) [57]. Akbari et al. described in their research performed on 305 patients with dyspepsia that the prevalence of migraine was significantly higher in female patients compared to male patients (48.9% vs. 35.5%, respectively, *p* < 0.010) [58]. This idea may be explained by the fact that girls are, firstly, usually more aware of their symptoms; secondly, they are prone to encounter more headache episodes along with the debut of menstrual cycles; and thirdly, females may present a more important bacterial load.

Regarding the area of living, the results of our study describe an increased incidence of headache/migraine among the pediatric population living in the rural area (80%) than those from the urban one (20%). These results are similar to the environmental distribution among the *H. pylori*-positive patients in our study, where 75.3% came from rural environments, whereas 24.7% lived in urban areas. These facts are consistent with Martin et al., who found that along with females and white people, the individuals residing in rural areas were more likely to suffer from headache than their respective comparison batches [59]. This hypothesis may be explained by the lack of specialist care in rural regions, thus leading to a lower adherence to headache or migraine management and treatment.

Knowing that *H. pylori* may lead to a low luminal pH by decreasing the bicarbonate secretion and increasing the acid secretion, this microorganism is recognized for its capacity to weaken the mucosa in the areas of gastric metaplasia and to make the mucosa more vulnerable to acid secretion. With many findings that associated the presence of *H. pylori* infection with migraine, Hormati et al. even postulated that a low value of the gastric pH can represent a trigger for headache, whereas the treatment with proton pump inhibitor (PPI) drugs may contribute to a significant improvement in both the severity and frequency of migraine attacks [57]. Another hypothesis raised by Lileikyte et al. involves the role of *H. pylori* in the development of vestibular migraine through irritation of the respiratory mucosa by the gastric acid, with subsequent inflammation or direct local infection caused by the presence of *H. pylori*, but these interesting affirmations need further explorations [60].

Further, individuals who suffer from migraines may have an increased risk of developing vitamin B12 deficiency due to the use of non-steroidal anti-inflammatory drugs for acute symptom relief and an increased incidence of active *H. pylori* infection [61]. In their article, Urits et al. describe that many individuals with migraines also experience gastro-intestinal damage, and *H. pylori* infection further impairs vitamin B12 absorption by destroying gastric parietal cells and reducing the availability of intrinsic factors [62].

Recently developed antimigraine drugs, such as anti-calcitonin gene-related peptide antibodies (CGRP) and monoclonal antibodies, offer a promising breakthrough in the treatment of migraine. The antimigraine mechanism of action of these drugs is similar to that of a kynurenic acid analogue, which can eliminate nitroglycerin-induced hyperalgesia by increasing CGRP expression. The kynurenine pathway, which is involved in the metabolism of L-tryptophan, is known to be altered in functional gastrointestinal diseases that are associated with migraines. In consequence, targeting this pathway may be an effective approach for treating both migraine and functional gastrointestinal diseases [63].

The present study showed a significant association between the infection with *H. pylori* and headache, but this relationship needs further studies. Although findings about the correlations between *H. pylori* and headache pathogenesis have been accumulating, the existing data do not completely amount to an unequivocal conclusion. However, there are certain effects of *H. pylori* infection, such as a decrease in food sensitivity, a lack of changes in plasma levels of thiobarbituric acid-reactive substances, and nitric oxide metabolites in infected patients compared to control subjects, and there is a similar prevalence of infection with *H. pylori* in patients with migraines compared to healthy subjects. These effects could be interpreted as valid arguments against *H. pylori* being considered a risk factor for migraines [36,47,53].

The need for establishing a definite association between headache and the *H. pylori* infection remains of great importance. Furthermore, our study has its limitations, such as a lack of paraclinical investigations consequent to the irregularity of the funding availability, the impossibility of performing real-time PCR for testing the antimicrobial susceptibility in *H. pylori* infection, along with the inability to conduct a long-term follow-up of the children due to their refractoriness.

## 5. Conclusions

Currently, there is sufficient evidence that correlates the increased frequency of migraine or headache with various gastro-intestinal disorders, compared to the general pediatric population. However, no clear association between *H. pylori* infection and headache was established to present. The gut–brain axis needs further exploration, as it is indicated in playing an important role between the *H. pylori* infection—migraine relationship.

It is important to note that further investigations should be carried out to evaluate the effectiveness of *H. pylori* eradication on the severity of headaches, the long-term clinical implications of this potential relationship, the assessment of multiple strains of *H. pylori* in children with headache, and the ethnicity of the participants under study. Furthermore, the variation in *H. pylori* in different regions should also be considered as a significant factor in future evaluations. We are certain that a better understanding of this association between headache and gastrointestinal disorders in children is of great clinical importance for both prompt diagnosis and treatment.

## Figures and Tables

**Table 1:** Study group presenting or not presenting *H. pylori* infection.

Infection with *H. pylori*		%
absent	1215	69.2
present	542	30.8
**Headache**		
absent	1627	92.6
present	130	7.4
**Sex**		
female	1210	68.9
male	547	31.1
**Area of living**		
urban	643	36.6
rural	1114	63.4

**Table 2:** Estimated parameters in testing the association between *H. pylori* infection and headache.

	Headache (+)	Headache (−)
**HP (+)**	54	488
**HP (−)**	76	1139
	*p* value = 0.006	

HP—*Helicobacter pylori*.

**Table 3:** Differences between sex-stratified subgroups.

	HP (+)	HP (−)	Headache (+)	Headache (−)
**Sex**				
male	145	402	22	525
female	397	813	108	1102
		*p* = 0.0002 chi-squared		

HP—*Helicobacter pylori*.

## Data Availability

The data presented in this study are available on request from the corresponding author.
